# A SARS-CoV-2 full genome sequence of the B.1.1 lineage sheds light on viral evolution in Sicily in late 2020

**DOI:** 10.3389/fpubh.2023.1098965

**Published:** 2023-01-26

**Authors:** Miguel Padilla-Blanco, Francesca Gucciardi, Vicente Rubio, Antonio Lastra, Teresa Lorenzo, Beatriz Ballester, Andrea González-Pastor, Veronica Veses, Giusi Macaluso, Chirag C. Sheth, Marina Pascual-Ortiz, Elisa Maiques, Consuelo Rubio-Guerri, Giuseppa Purpari, Annalisa Guercio

**Affiliations:** ^1^Departamento de Farmacia, Facultad de Ciencias de la Salud, Universidad Cardenal Herrera-CEU (UCH-CEU), CEU Universities, Valencia, Spain; ^2^Istituto Zooprofilattico Sperimentale della Sicilia “A. Mirri”, Palermo, Italy; ^3^Department of Genomics and Proteomics, Instituto de Biomedicina de Valencia del Consejo Superior de Investigaciones Científicas (IBV-CSIC) and Centre for Biomedical Network Research on Rare Diseases of the Instituto de Salud Carlos III (CIBERER-ISCIII), CEU Universities, Valencia, Spain; ^4^Departamento de Ciencias Biomédicas, Facultad de Ciencias de la Salud, Universidad Cardenal Herrera-CEU, CEU Universities, Valencia, Spain; ^5^Departamento de Medicina, Facultad de Ciencias de la Salud, Universidad Cardenal Herrera-CEU, CEU Universities, Valencia, Spain

**Keywords:** SARS-CoV-2, B.1.1 variant, new sequence, virus monitorization, Sicily, COVID-19, novel SARS-CoV-2 genome sequence

## Abstract

To investigate the influence of geographic constrains to mobility on SARS-CoV-2 circulation before the advent of vaccination, we recently characterized the occurrence in Sicily of viral lineages in the second pandemic wave (September to December 2020). Our data revealed wide prevalence of the then widespread through Europe B.1.177 variant, although some viral samples could not be classified with the limited Sanger sequencing tools used. A particularly interesting sample could not be fitted to a major variant then circulating in Europe and has been subjected here to full genome sequencing in an attempt to clarify its origin, lineage and relations with the seven full genome sequences deposited for that period in Sicily, hoping to provide clues on viral evolution. The obtained genome is unique (not present in databases). It hosts 20 single-base substitutions relative to the original Wuhan-Hu-1 sequence, 8 of them synonymous and the other 12 encoding 11 amino acid substitutions, all of them already reported one by one. They include four highly prevalent substitutions, NSP12:P323L, S:D614G, and N:R203K/G204R; the much less prevalent S:G181V, ORF3a:G49V and N:R209I changes; and the very rare mutations NSP3:L761I, NSP6:S106F, NSP8:S41F and NSP14:Y447H. GISAID labeled this genome as B.1.1 lineage, a lineage that appeared early on in the pandemic. Phylogenetic analysis also confirmed this lineage diagnosis. Comparison with the seven genome sequences deposited in late 2020 from Sicily revealed branching leading to B.1.177 in one branch and to Alpha in the other branch, and suggested a local origin for the S:G118V mutation.

## Introduction

**Figure 1:**
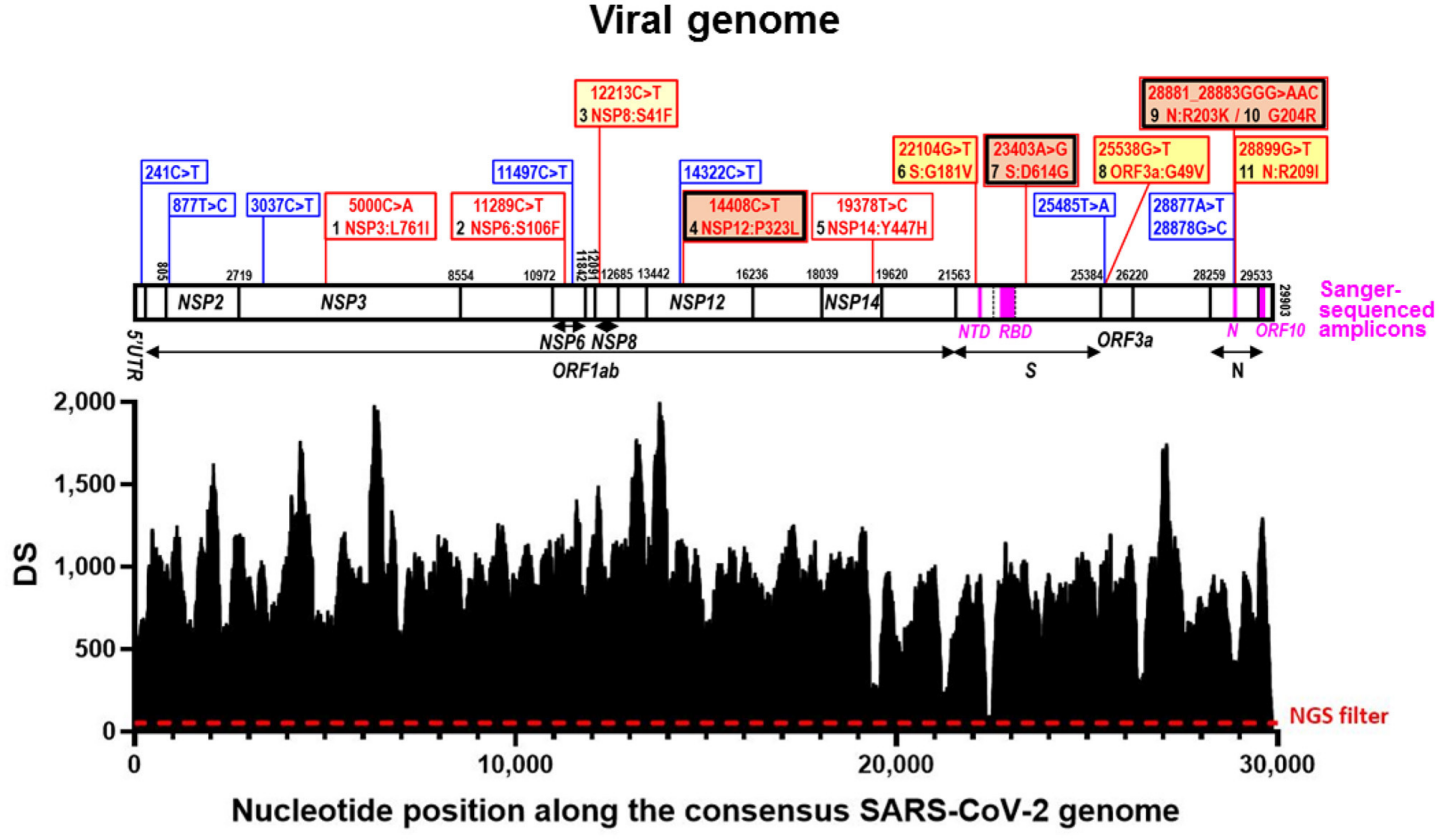
Deepness of sequencing (DS) along the viral genome and sequence variations identified. In the l*ower part*, SARS-CoV-2 genomic coverage obtained by next-generation sequencing (NGS). The horizontal axis corresponds to positions in the SARS-CoV-2 Wuhan-Hu-1 consensus genome sequence (GenBank ID: NC_045512.2) and the vertical one to the depth of the sequence (DS) at a given position. The dashed horizontal line marks the NGS filter (DS ≥ 50). *Above* this, aligned over the nucleotide positions, the viral genome is schematized as a horizontal bar, with identification of the genes hosting nucleotide substitutions in our reported genome. Gene boundaries are marked with vertical lines crossing the bar, giving their corresponding nucleotide position in the sequence. Gene names are inscribed into the bar or placed under it, with indication with horizontal short lines or double-pointed arrows, of their spans (including the one of *ORF1ab*, which is a polyprotein-encoding gene). The part of the *S* gene that encodes the Receptor Binding Domain (RBD) is marked within the genome bar using transversal broken lines to indicate its boundaries. The amplicons used in Sanger sequencing (Table 1) are marked as magenta-colored rectangles within the genome bar, with their abbreviated names given below in magenta. Blue and red vertical lines emerging up from the genome bar, respectively, mark synonymous and non-synonymous sequence variants, described in the corresponding banners (equally colored). For non-synonymous changes the banners inscribe the nucleotide change (nucleotide numbering is that for the complete genome) and the amino acid substitution (amino acid numbering is that for the sequence of the mature protein), also numbering in black the amino acid changes from 1 to 11 as they occur along the genome from 5′ to 3′. A double frame in black and red indicates that the amino acid change belongs to the canonical set for the B.1.1. lineage. The background color of the banner is orange for amino acid changes reported in millions of sequences, deep yellow for tens of thousands, light yellow for thousands, and white for smaller numbers. These numbers are given in Table 2.

From the time of its emergence in late 2019 in China (1) and along the nearly 3 years of pandemic with a toll of >635 million cases and > 6.5 million deaths (2), the SARS-CoV-2 virus has evolved, accumulating mutations in its genome. This genome is a positive-sense single-stranded RNA (+ssRNA) that encodes four structural proteins (spike, S; envelope, E; matrix, M; and nucleocapsid, N), sixteen non-structural proteins (NSP1 to NSP16, all derived from the *ORF1ab* gene), and nine putative accessory factors (ORFs 3a, 3b, 6, 7a, 7b, 8, 9b, 9c, and 10) (3) (see Figure 1 for a non-exhaustive scheme of the viral genome). Insertions, deletions, and particularly amino acid substitutions in the encoded proteins are the main actors of viral evolution, a process which is largely shaped by selection toward increased viral transmissibility, fitness and escape from antiviral challenges (antibodies and vaccines) (4). The D614G amino acid substitution in the S protein was one of the first changes that succeeded in being fixed in subsequent viral lineages since it increased viral transmissibility (5, 6) (reported up-to day in >13 million genome sequences deposited in GISAID, Figure 1). In the summer of 2020, the B.1.177 variant spreading from Spain incorporated another mutation, A222V, in the viral S protein, a change generally considered neutral (7), although such view might be challenged by the repeated events of independent re-emergence of this mutation (8). Variants B.1.160 and B.1.258 appeared approximately simultaneously with the B.1.177 variant and coexisted with it in some countries, (7, 9). They, respectively, hosted in the S protein the S477N and N439K substitutions (4), both of them mapping in the receptor binding domain (RBD) of the S protein, the part of S that mediates virus attachment to its cellular receptor (the angiotensing-converting enzyme 2, ACE2). Both mutations apparently enhanced slightly the affinity of the RBD for ACE2 (10), although these variants did not outperform B.1.177. Since then, and particularly after November 2020, new SARS-CoV-2 variants have appeared with significantly increased fitness relative to previous variants, not only in terms of transmissibility, but also in terms of evasion of neutralizing antibodies and vaccine-mediated immunity (11). Presently the World Health Organization (WHO) designates variants as Variants Being Monitored (VBMs), Variants of Interest (VOIs; presently none) or of Concern (VOCs, three in October 27, 2022, all from the Omicron lineage) for which there is either potentiality (VOIs) or clear evidence (VOCs) for increased transmissibility, severity or escape from diagnosis or from antibodies/vaccination; and, finally, Variants of High Consequence (VOHCs, presently none) which are of obligatory declaration and that are really those causing a severe and highly dangerous pandemic or highly epidemic situation [(12); and https://www.ecdc.europa.eu/en/covid-19/variants-concern].

As a part of the effort to monitor novel viral variants (13), we report here a unique variant identified by us in a retrospective study of samples collected in December 2020 in the island of Sicily (14). Among ~60 samples analyzed, a sample revealed a novel variant that we feel merits report, as it adds substantial information in an insular area (Sicily) that hosts a relatively large population (~5 million inhabitants) for which whole genome sequencing studies for the period of sample collection (September to December 2020) were extremely scarce (seven found in our search of GISAID: EPI_ISL_ values 2308744, 2308745, 2308746, 2308747, 2308749, 3274295, and 910332) despite the fact that >85,000 cases of COVID-19 had been diagnosed in the island by December 2020 (14). Monitoring of viral variants is an important task not only for tracing viral evolution, but also for health reasons that include the development of appropriate fast diagnostic tools and to anticipate possible vaccine evasion events in emerging variants. In addition to VOIs and VOCs, minority variants also are relevant, especially in terms of virus monitorization. In fact, some of the substitutions described for less prevalent variants have later on been reported in VOCs (13), some of them *via* independent events of re-emergence as observed for the A222V mutation in the S protein (8).

**Table 1:** Nucleotide and amino acid substitutions found after Sanger sequencing of the four PCR-amplified viral genomic regions.

**qPCR reaction**	**Genomic target region/amplicon^a^**	**Nucleotide substitution^b^**	**Amino acid substitution^c^**	**GenBank accession number**	**Amino acid substitution searched for^c^**	**Known SARS-CoV-2 variants that could be detected**
1	*S* (*NTD*)/22160-22239	None found	None	OP618232	S:A222V	B.1.177
2	*N/*28871-28964	28877_28878 AG > TC	Synonymous	OP620391	N:A220V	
		28881_28882 GG > AA	N:R203K			
		28883 G > C	N:G204R			
		28899 G > T	N:R209I			
3	*ORF10/*29558-29704	None found	None	OP618233	ORF10:V30L	
4	*S* (*RBD*)/22728-23124	None found	None	OP618234	S:N501Y	B.1.1.7 (Alpha)
					S:K417T S:E484K S:N501Y	P.1 (Gamma)

The sequences obtained have been submitted to GenBank with the Accession Numbers given (column 5). The qPCR reactions were carried out to detect the changes causing the amino acid substitutions indicated in column 6 in order to identify the SARS-CoV-2 variants indicated in the last column.

^a^Figures refer to nucleotide positions in the whole genome reference sequence NC_0455.12.

^b^Substitution relative to the base in the consensus genome. As the sequencing was based on the genome retrotranscribed to DNA, thymine should correspond to uracil in the viral RNA genome.

^c^Amino acid positions are those for the encoded protein.

**Table 2:** Base substitutions found by NGS of the viral genome, and corresponding amino acid substitutions, indicating the abundance and frequency of each amino acid change among the genome sequences deposited in the GISAID database on December 4, 2020 and, after the slash, on November 2, 2022.

**Base change^a^**	**Gene**	**Encoding mature protein**	**Amino acid substitution** ^**b**^				**¿Change present in canonical B.1.1?**
			**No**.	**Change**	**Abundance in GISAID^c^**	**Frequency in GISAID (%)^c^**	
241 C > T	*5′ UTR*	–	–	–	NT	NT	NO
877 T > C	*ORF1ab*	NSP2	–	–	NT	NT	NO
3037 C > T		NSP3	–	–	NT	NT	NO
5000 C > A			1	NSP3:L761I	19/1,196	0.003/0.009	NO
11289 C > T		NSP6	2	NSP6:S106F	180/847	0.032/0.006	NO
11497 C > T			–	–	NT	NT	NO
12213 C > T		NSP8	3	NSP:S41F	208/3,111	0.037/0.023	NO
14322 C > T		NSP12	–	–	NT	NT	NO
14408 C > T			4	NSP12:P323L	0.5/13.3 ( × 10^6^)	93.21/97.25	YES
19378 T > C		NSP14	5	NSP14:Y447H	15/556	0.003/0.004	NO
22104 G > T	*S*	S	6	S:G181V	758/40,256	0.135/0.295	NO
23403 A > G			7	S:D614G	0.5/13.3 ( × 10^6^)	93.96/99.19	YES
25485 T > A	*ORF3a*	ORF3a	–	–	NT	NT	NO
25538 G > T			8	ORF3a:G49V	229/38,469	0.041/0.283	NO
28877 A > T	*N*	N	–	–	NT	NT	NO
28878 G > C^d^			–	–	NT	NT	NO
28881 G > A^d^			9	N:R203K	0.16/7.6 ( × 10^6^)	29.55/56.27	YES
28882 G > A^d^							
28883 G > C^d^			10	N:G204R	0.16/7.6 ( × 10^6^)	29.30/55.72	YES
28899 G > T^d^			11	N:R209I	2,693/12,880	0.481/0.094	NO

^a^Nucleotide position, and substitution relative to the consensus SARS-CoV-2 Wuhan-Hu-1 genome (GenBank NC_045512.2). As the sequencing was based on the genome retrotranscribed to DNA, thymine should correspond to uracil in the viral RNA genome.

^b^Amino acid positions are those for the mature proteins.

^c^On December 4, 2020/November 2, 2022. NT, not tested.

^d^Detected by both Sanger sequencing of amplicons and by NGS of the whole genome.

The unique SARS-CoV-2 genome sequence reported here was first identified by us by Sanger sequencing of limited regions of the genome, and then was fully confirmed as novel by next generation sequencing (NGS) of 99.7% of the viral genome. We found that the genomic sequence of this virus derives from a B.1.1 variant, but that it diverts from the canonical blueprint for this variant in an important number of changes. Thus, the viral genomic sequence reported here hosts 20 single-base substitutions (two on two adjacent bases of the same codon) resulting in 11 amino acid substitutions, of which only 4 are present in the canonical B.1.1 variant. Furthermore, the particular combination of genome sequence changes reported here has not been identified until now. Relations of the genomic sequence with the few sequences reported for that period in Sicily, and with other full genomic viral sequences are also reported here.

## Methods

### Sample procurement, processing and molecular techniques including Sanger sequencing of selected-regions, and whole-genome sequencing

The nasopharyngeal swab sample in which the present viral variant was found was procured in Palermo (Sicily, Italy) by the Virology Department of the Istituto Zooprofilattico Sperimentale della Sicilia “A. Mirri” (Palermo, Italy). This is a diagnostic center of SARS-CoV-2 and other emerging viruses that was routinely collecting nasopharyngeal swab samples for the detection of SARS-CoV-2. The collection date of our study's sample was December 4, 2020. The total RNA of our study's sample was positive for SARS-CoV-2 infection using a commercial real-time reverse transcription polymerase chain reaction assay (rRT-PCR) (14). It was sent, preserved in dry ice, for detailed molecular studies to the Health Sciences Faculty of UCH-CEU University (Valencia, Spain).

All steps involved in the molecular studies leading to Sanger sequencing of selected amplicons (Figure 1) have been reported previously by us (14). In short, following retrotranscription to cDNA, four qPCR assays were routinely carried out that provided a shortcut to typing the SARS-CoV-2 virus by using very limited automated Sanger sequencing of the four amplicons (bases 22160-22239 and 22728-23124 of the *S* gene, the longest of the two encoding part of the RBD; and bases 28871-28964 and 29558-29704 from the *N* and *ORF10* genes, respectively, Table 1). The amplicons were sequenced in a core gene sequencing facility (Príncipe Felipe Research Center of Valencia, Spain) using an ABI Prism 3730 sequencer, from Applied Biosystems (Foster City, CA, USA).

As the analysis of the sequences obtained in the Sanger approach using the Bioedit ver. 7.2.5 software (15) suggested a novel viral variant, we performed Next Generation Sequencing (NGS) of the entire viral genome by the approach described in detail in (16), in which Sequencing Multiplex SL (Valencia, Spain; a spinoff of the Health Research Institute of the Hospital Clínico de Valencia, INCLIVA) utilized Illumina NGS on cDNA retrotranscribed from the total RNA in our sample using random hexameric oligonucleotides and SuperScript IV reverse transcriptase (ThermoFisher). With this approach, 516,308 reads were obtained, and were submitted to the NCBI Sequence Read Archive (SRA) repository, under the BioProject accession number PRJNA900410. For analysis and reconstruction of the whole genome sequence, the Galaxy platform (17) was used to carry out the bioinformatic analysis of the raw reads. Quality of reads was visualized using FASTQC and, using the Cutadapt software, reads with less than Q20 of quality and smaller size than 100 bp were removed, yielding 307,219 high quality sequences mapping to the SARS-CoV-2 consensus genome (Figure 1). These reads were aligned against the SARS-CoV-2 reference genome using the Burrows-Wheeler algorithm (BWA-MEM), obtaining the corresponding aligned file in BAM format, which was converted to the respective SAM binary file utilizing SAMtools. Finally, by the combination of SAMtools and BCFtools, we obtained a VCF file which contained not only the genomic positions which appeared mutated in our sample, but also the depth sequence (DS, number of times each genomic position was read by sequencing) of the vast majority of SARS-CoV-2 genomic positions. Positions which presented a DS higher than 50 and which were different to the consensus sequence in at least 75% of the reads that covered these positions were classified as mutated ones. Furthermore, the presence of these mutated positions was visualized using the Integrative Genomics Viewer (IGV) (18).

The final aligned sequence covered 99.7% of the entire viral genome, with only 90 positions of the 29,903 nucleotides of the whole reference viral genome (GenBank identifier NC_045512.2) not properly covered (positions 1–39 and 29,853–29,903) (Figure 1).

The obtained sequence was submitted to the GISAID database (19) (Accession ID: EPI_ISL_13157456). The sequence was used for BLASTN analysis (20) of GISAID and NCBI databases to test whether the obtained sequence had been reported previously. It was aligned with the consensus genome sequence and with the most relevant SARS-CoV-2 variants (including past and present VOCs). Following the alignment conducted with MAFFT aligner version 7 (21), aligned sequences were subjected to phylogenetic analysis using the MEGA11 software (22), as previously reported (16). In brief, distance matrices were calculated, and tree topology was inferred by the maximum likelihood method based on p-distances (bootstrap on 2,000 replicates, generated with a random seed). This process was done, too, with the seven viral sequences from Sicily deposited in GISAID for the period of September to December of 2020.

## Results

As a shortcut to differentiate among major pre-existing variants, and particularly to identify B.1.177, a variant which was highly prevalent in Europe at the time of collection of this sample (7), we first PCR-amplified and Sanger-sequenced four genomic regions (Figure 1 horizontal bar; and Table 1). Analysis of three regions corresponding to the part of the *S* gene that encodes the N-terminal domain (NTD) of the S protein and two regions within the coding sequences of the *N* and *ORF10* genes (Table 1) did not uncover any characteristic B.1.177 substitutions, revealing instead in the *N* gene six nucleotide substitutions clustered in a group of two adjacent ones (affecting the same codon and constituting a synonymous change); in another group of three adjacent ones (affecting two successive codons, causing the two amino acids substitution R203K/G204R in the N protein) and a third isolated substitution also causing a non-synonymous change in the N protein (R209I). The R203K and G204R substitutions in the N protein are observed in the Alpha (B.1.1.7) and Gamma (P.1) variants, two variants that in December 2020 were increasing their prevalence and were of concern (9). For differentiation between Alpha and Gamma variants we used the fourth Sanger-sequenced amplicon. This amplicon encompasses a central region of the *S* gene that encodes a part of the receptor binding domain (RBD) of the S protein. Alpha and Gamma variants contain therein a unique combination of marker substitutions (Table 1) (9). To our surprise, we did not find in this amplicon any amino acid substitutions characteristic of these variants, failing to support the identification of our viral sample as belonging to the Alpha, Gamma or to the B.1.177 lineages. This strongly suggested that the virus in this sample represented a different, perhaps new variant, prompting us to use whole-genome sequencing by NGS to try to characterize it.

**Figure 2:**
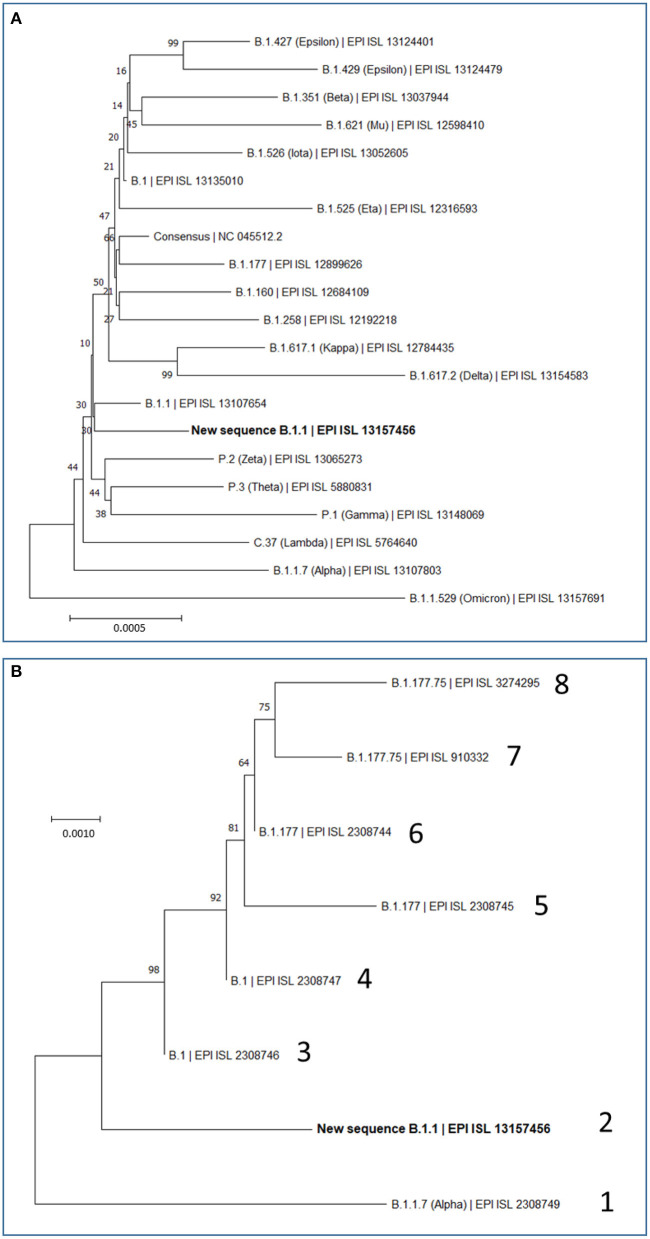
Maximum Likelihood Trees based on complete SARS-CoV-2 genomes. Evolutionary analyses were conducted in MEGA (see Methods), inferring the histories using the neighbor-joining method. Optimal trees are shown. The percentage of replicate trees in which the associated taxa clustered together is shown next to the branches. The scale bars measure phylogenetic distances as the indicated nucleotide substitutions per site. Optimal trees are shown. Trees are drawn to scale, with branch length in the same units as those of the evolutionary distance used to infer the phylogenetic tree. **(A)** Phylogenetic relations of our new B.1.1 sequence in the context of the most relevant SARS-CoV-2 variants, including variants which during the pandemic have been considered at least once as VOCs (Alpha, Beta, Gamma, Delta and Omicron) or VOIs (Epsilon, Mu, Iota, Eta, Kappa, Zeta, Theta, and Lambda), and also including the most prevalent variants (B.1, B.1.177, B.1.160, and B.1.258) that were circulating in Europe before the collection date of our study's sample. This analysis involved 21 nucleotide sequences, with a total of 29,919 position in the final dataset. **(B)** Phylogenetic relations of our new B.1.1 sequence with the seven previously reported SARS-CoV-2 sequences from Sicily in the time spanning between September and December 2020. Sequence lineage and GISAID ID are given for each genome, giving it an arbitrary number (1–8). Our new B.1.1 sequence is highlighted in bold in **(A, B)**.

Of the 29,903 nucleotides of the complete reference sequence of the SARS-CoV-2 genome, our NGS approach generated high-quality sequence of appropriate deepness (see Methods) for nucleotides 40 to 29,853, corresponding to 99.7 % of the whole genome viral reference sequence (Figure 1). The sequence was assembled with appropriate bioinformatics tools (see Methods), identifying 20 base substitutions relative to the reference sequence, of which 12 were nonsynonymous substitutions (11 amino acid substitutions) and 8 were synonymous changes (Figure 1 and Table 2). Based on the SARS-CoV-2 variant classification of GISAID, the virus was automatically assigned by the GISAID server to the B.1.1 lineage, a European lineage traced back to the 4th of January of 2020 with 3 clear single nucleotide substitutions, 28881 G>A, 28882 G>A and 28883 G>C (23), all three present in our genomic sequence (Figure 1). The COV-LINEAGES organization webpage, visited on November 1, 2022 (23), gives 22,790 and 50,400 viral genomes designated and adscribed, respectively, to the B.1.1 lineage. Our sequence carries four of the five changes given in (23) as present in at least 75% of the sequences corresponding to the B.1.1 lineage: NSP12:P323L (equivalent to ORF1b:P314L; mapping in the NSP12 protein), S:D614G (S protein), and N:R203K and N:G204R (N protein) (Figure 1 and Table 2, banners with an inner black frame), only lacking the lineage marker mutation ORF8:S84L (ORF8 protein). The occurrence in our genomic sequence of four of the 5 marker sequence changes shared by 75% of the viral isolates ascribed to the B.1.1 lineage reinforce the adscription of our viral sample to that lineage. This adscription is supported, too, by the results of phylogenetic analysis that showed (Figure 2A) that our genome sequence clusters together with B.1.1 viral sequences (for simplicity, only one shown in Figure 2A).

In addition to the presence of four of the five “marker” mutations of the lineage, our genome sequence revealed seven additional non-synonymous sequence variations that are not characteristic of the B.1.1 lineage and that affect proteins NSP3, NSP6, NSP8, NSP14, S, ORF3a and N (abbreviated as mutations 1, 2, 3, 5, 6, 8, and 11, respectively; Figure 1 and Table 2). None of these amino acid changes was unreported, as shown by their occurrence in sequences previously deposited in the GISAID database (Table 2). All of them had already been reported by the date of collection of our sample in early December 2020 (Table 2), although two of them, 1 and 5 (affecting NSP3 and NSP14, respectively), had only been reported in very few sequences (< 20 genomes) (Table 2). None of these two variants as well as the non-synonymous changes 2 (in NSP6) and 3 (in NSP8) has spread much, having been reported in < 0.025% of the >13.6 million viral genomic sequences now deposited in GISAID. This low frequency does not support a substantial effect of these mutations on viral fitness. Variants 6, 8, and 11 (affecting S, ORF3a and N proteins, respectively; Figure 1, banners in deep yellow color) appear to have been somewhat more successful in spreading, as they have been found in, respectively, 40,256, 38,461, and 12,880 viral genomes deposited in GISAID. Only 11 genomic sequences deposited up to now in GISAID combine these three last sequence variants with the four highly prevalent sequence variants (sequence variants in orange and in deep yellow in Figure 1, giving the combination of variants, 4, 6, 7, 8, 9, 10, and 11). This occurrence is lower (8 instances) when mutation 1 is added to the above set of non-synonymous substitutions. Addition of any other of the three remaining variants, 2, 3, or 5, reduces the number of sequences to 1, our own sequence, that thus, it is unique in the database. This last result agrees with our independent BLASTN analysis carried out with the entire databases of full genome sequences deposited in GISAID and GenBank, which failed to identify any sequence with the full set of non-synonymous mutations identified in our genomic sequence.

We also investigated by phylogenetic analysis (Figure 2B) the degree of closeness of this novel viral genome sequence with the other seven SARS-CoV-2 genomic sequences reported in the period of September to December of 2020. Two of these genomes (labeled 3 and 4 in Figure 2B) belong to the B.1 lineage and can be considered the stem from which all other sequences in this group of eight sequences have evolved. This diversification occurred in two separate branches (Figure 2B). One branch encompasses four viral isolates (sequences labeled 5, 6, 7, and 8 in Figure 2B), which belong to the B.1.177 and B.1.177.75 lineages. The other branch encompasses the viral genome reported here and the one labeled 1 in Figure 2B, respectively belonging to B.1.1 and B.1.1.7 (Alpha) lineages. This phylogenic tree, which is based on the alignment of the entire genome sequences, thus taking into consideration synonymous and non-synonymous sequence changes, is confirmed by just looking at the non-synonymous changes observed in these eight sequences (Supplementary Table 1). Only two amino acid changes, NSP12:P323L and S:D614G, are common to all eight sequences of Figure 2B. The top branch exhibits the S:A222V sequence that was very prevalent in Europe after the summer of 2020, having apparently spread from Spain, while the lower branch exhibits the characteristic N:R203K and N:G204R variants that emerged much earlier, in January 2020, in the B.1.1 lineage.

## Discussion

This study characterizes a novel SARS-CoV-2 variant that occurred in Sicily in the second wave of COVID-19, when the B.1.177 variant was the predominating lineage circulating through the island (14). Our novel variant has not been reported anywhere else, judged from its lack of deposit into the GISAID and GenBank databases, but it can be classified as belonging to lineage B.1.1. This last lineage had emerged very early on in the pandemic and incorporated the most successful [in terms of spreading (24)] combination of non-synonymous variants NSP12:P323L and S:D614G (Table 2). The mechanism of the effects of the NSP12 protein mutations P323L that led it to cosegregate with the S protein mutation D614G remain uncertain. NSP12 is the catalytic, major component of RNA-dependent RNA polymerase (RdRp) (25, 26), and the P323L substitution maps in the interaction domain of this polymerase (27). It is tempting to speculate that this mutation could favor the emergence of novel viral variants by impairing the 3′ to 5′ exonuclease proofreading activity of RdRp thus decreasing replication fidelity. This would agree with the observation of rapid generation of novel variants that occurred after the B.1 variant emerged and that accelerated later on (see the Introduction), although it is true that the appearance of novel variants must have also been favored by the increase of the number of infections occurring worldwide as COVID-19 crossed country boundaries and became a pandemic. In addition, this mutation may have helped the virus escape from antibodies raised in patients previously infected by the original Wuhan variant (28). This advantage should not be diminished by the fact that remdesivir, widely used in the treatment of severe COVID-19, appears to exhibit more affinity for the mutant form of RdRp than for the previous form of this enzyme not hosting this mutation (29), since only a minority of the infected population is treated with remdesivir (possibly < 10% of the infected people).

In any case, our variant characterization and its comparison with the other seven viral specimens characterized in Sicily by having their whole genome sequenced in late 2020, help recreate a scenario in which two separating lineage branches were largely imported. Nevertheless, our viral isolate might attest some degree of local evolution toward the Alpha (B.1.1.7) lineage represented in one of the 8 sequenced genomes studied from late 2020 (Figure 2B). The uniqueness of the sequence reported here suggests some limited local evolution from the imported parental B.1.1 lineage. Whichever the level of local evolution, the main message of the observation of these 8 fully characterized Sicilian viral sequences is that variants roaming though continental Europe were also circulating in the island. This agrees with our previous conclusion based on the study of 54 viral samples obtained in Palermo (14), that isolation due to the insularity of Sicily was not an important factor, at least for the second-wave period of the pandemic, a period that did not show founder effects that could reveal the potential isolation due to insularity. Nevertheless, given the fact that S protein mutations are particularly prone to impact on virus fitness and on its escape from pre-existing antibodies (30), it is interesting that, of the two S protein amino acid variants observed in our viral genome, the G181V substitution has had modest but substantial success (Table 2) and appears to have a local origin. Thus, this mutation was found in the second half of year 2021 in 7 of 252 genotypes of the then dominant Delta lineage in the Italian mainland region of Calabria (31), which is the closest part of peninsular Italy to Sicily (from which it is separated only by the Messina straight) supporting the regional origin of this mutation. These facts render desirable the experimental investigation of the effects of this mutation on viral infectivity and sensitivity to antibodies.

Also interesting for its potential consequences is the G49V mutation in ORF3a, since G49 sits in the center of the hydrophobic cluster that seals the pore formed by the homodimer of this channel protein (Protein Databank entry 7KJR, https://www.rcsb.org/structure/7KJR) (32). ORF3a is emerging as a key element in the pathogenicity of COVID-19, mediating apoptotic and autophagy-related proinflammatory effects of the viral infection (33, 34) possibly by allowing Ca^2+^influx into the cell, as this cation has been shown to be channeled across membranes by the ORF3a dimer (32). The ORF3a transmembrane pore is sealed at membrane level by a hydrophobic patch of residues that glues together its six (three per subunit) transmembrane helices. This patch centrally includes G49 (belonging to transmembrane helix 1). The replacement by valine of G49 in the G49V mutant might distort the 6-helix bundle of the dimer that constitutes the closed pore, possibly increasing the frequency of the opening and Ca^2+^ passage, thus increasing disease severity by enhancing the proinflammatory and apoptotic potential of the virus. This possibility, and the modest but substantial success (Table 2) of this ORF3a variant, possibly justifies the experimental testing of the consequences of this amino acid substitution.

Of the other substitutions in our viral genome, the NSP12:P323L and S:D614G mutations are well-known for being epidemiologically very successful (24) but also for having been found more frequently in patients suffering severe COVID-19 than in those patients with milder COVID-19 (35). Similarly, the epidemiologically highly successful double substitution in the N protein R203K/G204R, has been reported to increase viral infectivity, fitness, and virulence and to promote a subgenomic RNA promoting recombination (36, 37). These effects of the N:R203K/G204R mutations might be further enhanced in the present virus by the coexistence of N:R209I, a drastic amino acid substitution just five amino acids downstream from the N:G204R mutation.

In contrast to the above-mentioned substitutions, amino acid changes 1, 2, 3, and 5 appear of little concern, given their very low representation in the mutational universe of SARS-CoV-2. In particular, the L761I substitution in NSP3 is chemically trivial and thus probably neutral, and the S106F substitution in NSP6, although chemically drastic, affects a site that may be tolerant to sequence variation since S106 sits in an extracellular loop of the NSP6 membrane protein (38) in a site of lesser conservation where it is flanked by large hydrophobic residues (L**S**GF; hydrophobic residues underlined; S in bold-type).

In conclusion, this study retrospectively characterizes a unique viral variant found in Sicily in late 2020 that belongs to the B.1.1 lineage but which also exhibits a relatively large constellation of amino acid substitutions additionally to lineage marker mutations. Our sequence exemplifies the convenience of continuous monitoring SARS-CoV-2 sequences to understand virus evolution. This provides much needed information for a period and location for which the circulating viral lineages were very scarcely explored. The comparison of this genome sequence with the few other whole genome sequences of SARS-CoV-2 obtained at the same location and time supports a dynamic interaction of the island with continental Italy and Europe, but does not exclude some contribution of founder effects, exemplified here in an S protein mutation found in significant but not majority ratios 6 months later in Calabria, just across the Messina straight.

## Data availability statement

The datasets presented in this study can be found in online repositories. The name of the repository and accession number can be found below: NCBI Sequence Read Archive; PRJNA900410.

## Ethics statement

The studies involving human participants were reviewed and approved by Ethics Committee of Cardenal Herrera CEU University, Valencia, Spain (no. CEI20/083 released on 10/09/2020), and it is in agreement with the Helsinki Declaration.

## Author contributions

VR, EM, CR-G, GP, and AG conceived the study. FG, AL, GM, GP, and AG performed molecular SARS-CoV-2 detection. MP-B, TL, BB, and AG-P did the molecular variant characterization and bioinformatic analysis. MP-B, VV, CS, and MP-O performed the phylogenetic analysis. MP-B and VR did the GISAID work. MP-B, BB, VR, EM, and CR-G analyzed the results. MP-B, FG, BB, VR, EM, CR-G, GP, and AG were responsible for writing the paper. All authors contributed to this task, making substantial intellectual contributions, and having read, corrected, and approved the manuscript.
