# Supplementary material for: A SARS-CoV-2 full genome sequence of the B.1.1 lineage sheds light on viral evolution in Sicily in late 2020
Source: Front Public Health. 2023 Jan 26;11:1098965. doi: 10.3389/fpubh.2023.1098965 (PMC9909176; doi:10.3389/fpubh.2023.1098965)
Supplement: Supplementary file 1 [file Table_1.DOCX]

**Supplementary Table 1. Amino acid substitutions encoded by the genomic sequences from Sicily for the period of September to December of 2020.** The sequence identifiers are the ones given in Fig. 2B. The genome sequence reported here is highlighted in red. The amino acid changes encoded in each sequence are indicated by crosses and by shadowing yellow the corresponding cell.

| **Genome** | | | | | | | | | |
| --- | --- | --- | --- | --- | --- | --- | --- | --- | --- |
| **#** | | **1** | **2** | **3** | **4** | **6** | **5** | **7** | **8** |
| **GISAID identifier** | | **EPI_ISL**  **_2308749** | **EPI_ISL**  **_13157456** | **EPI_ISL**  **_2308746** | **EPI_ISL**  **_2308747** | **EPI_ISL**  **_2308744** | **EPI_ISL**  **_2308745** | **EPI_ISL**  **_910332** | **EPI_ISL**  **_3274295** |
| **Lineage** | | **B.1.1.7** | **B.1.1** | **B.1.** | **B.1.** | **B.1.177** | **B.1.177** | **B.1.177.75** | **B.1.177.75** |
| **Protein** | **Amino acid substitution** |  |  |  |  |  |  |  |  |
| NSP12 | P323L | + | + | + | + | + | + | + | + |
| S | D614G | + | + | + | + | + | + | + | + |
| S | A222V |  |  |  |  | + | + | + | + |
| N | A220V |  |  |  |  | + | + | + | + |
| NSP3 | P278S |  |  |  |  |  | + |  |  |
| NSP6 | A54S |  |  |  |  |  | + |  |  |
| S | Q675H |  |  |  |  |  | + |  |  |
| S | K1073N |  |  |  |  |  | + |  |  |
| 8 | P30L |  |  |  |  |  | + |  |  |
| NSP3 | T133I | + |  |  |  |  |  | + |  |
| S | Y144F |  |  |  |  |  |  | + | + |
| NSP2 | Q496H |  |  |  |  |  |  |  | + |
| NSP4 | F64L |  |  |  |  |  |  |  | + |
| NSP15 | V9F |  |  |  |  |  |  |  | + |
| NSP3 | L761I |  | + |  |  |  |  |  |  |
| NSP6 | S106F |  | + |  |  |  |  |  |  |
| NSP8 | S41F |  | + |  |  |  |  |  |  |
| NSP14 | Y447H |  | + |  |  |  |  |  |  |
| S | G181V |  | + |  |  |  |  |  |  |
| 3a | G49V |  | + |  |  |  |  |  |  |
| N | R203K | + | + |  |  |  |  |  |  |
| N | G204R | + | + |  |  |  |  |  |  |
| N | R209I |  | + |  |  |  |  |  |  |
| NSP3 | G307C | + |  |  |  |  |  |  |  |
| NSP3 | A890D | + |  |  |  |  |  |  |  |
| NSP6 | del^106^SGF^108^ | + |  |  |  |  |  |  |  |
| NSP10 | T12I | + |  |  |  |  |  |  |  |
| NSP12 | A716V | + |  |  |  |  |  |  |  |
| S | Del^69^HV^70^ | + |  |  |  |  |  |  |  |
| S | N501Y | + |  |  |  |  |  |  |  |
| S | A570D | + |  |  |  |  |  |  |  |
| S | P681H | + |  |  |  |  |  |  |  |
| S | T716I | + |  |  |  |  |  |  |  |
| S | D1118H | + |  |  |  |  |  |  |  |
| 8 | Q27STOP | + |  |  |  |  |  |  |  |
| N | D3L | + |  |  |  |  |  |  |  |
| N | L139F | + |  |  |  |  |  |  |  |
| N | S235F | + |  |  |  |  |  |  |  |
